# Carbapenem Usage in the Initial Antibiotic Therapy of Sepsis in Japanese Intensive Care Units

**DOI:** 10.7759/cureus.77271

**Published:** 2025-01-11

**Authors:** Eri Kobayashi, Atsushi Shiraishi, Toshiyuki Karumai, Yoshiro Hayashi, Toshikazu Abe, Hiroshi Ogura, Kushimoto Shigeki, Satoshi Gando, Kohji Okamoto, Yutaka Umemura, Junichi Sasaki, Yasukazu Shiino, Toshihiko Mayumi

**Affiliations:** 1 Department of Emergency and Critical Care, Saiseikai Utsunomiya Hospital, Utsunomiya, JPN; 2 Emergency and Trauma Center, Kameda Medical Center, Kamogawa, JPN; 3 Department of Intensive Care Medicine, Kameda Medical Center, Kamogawa, JPN; 4 Department of Emergency and Critical Care Medicine, Tsukuba Memorial Hospital, Ibaraki, JPN; 5 Department of Traumatology and Acute Critical Medicine, Osaka University Graduate School of Medicine, Osaka, JPN; 6 Division of Emergency and Critical Care Medicine, Tohoku University Graduate School of Medicine, Sendai, JPN; 7 Division of Acute and Critical Care Medicine, Department of Anesthesiology and Critical Care Medicine, Hokkaido University Faculty of Medicine, Sapporo, JPN; 8 Department of Surgery, Center for Gastroenterology and Liver Disease, Kitakyushu City Yahata Hospital, Kitakyushu, JPN; 9 Department of Emergency and Critical Care Medicine, Keio University School of Medicine, Shinjuku, JPN; 10 Department of Acute Medicine, Kawasaki Medical School, Okayama, JPN; 11 Department of Emergency Medicine, School of Medicine, University of Occupational and Environmental Health, Kitakyushu, JPN

**Keywords:** antimicrobial stewardship, carbapenem use, empiric therapy, epidemiology, inter-centre variation, sepsis

## Abstract

Background

Achieving a balance between the utilization and limitation of carbapenems for initial sepsis therapy is imperative, yet documentation on the use of carbapenems in sepsis treatment is limited. This study aimed to ascertain the prevalence of carbapenem use in Japanese intensive care units (ICUs) and evaluate the association between its use in the initial antibiotic therapy for sepsis and patient prognosis.

Methods

This study is a post hoc descriptive analysis of the Focused Outcome Research on Emergency Care for Acute Respiratory Distress Syndrome, Sepsis, and Trauma (FORECAST), a multicenter, prospective cohort study conducted in 59 ICUs in Japan from January 2016 to March 2017. This study described the rate of carbapenem use overall and in each ICU and assessed the association between carbapenem use and in-hospital mortality by generalized linear mixed effect model adjusting for patient characteristics as fixed effect confounders and the hospitals as random effect confounders.

Results

Out of 1140 participants, 627(55.0%) received and 513 (45.0%) did not receive carbapenems following the diagnosis of severe sepsis or septic shock. Patient severity was similar in both groups in terms of Sepsis-related Organ Failure Assessment (SOFA; 9 and 8) and Acute Physiology and Chronic Health Evaluation II (APACHE II; 23 and 22) scores. Among 48 of the 59 ICUs that registered more than three patients in the FORECAST registry, the median carbapenem utilization rate stood at 55.2% (minimum: 20.0%, maximum: 100.0%, IQR: 43.6%-67.2%). Hospital mortality rates were 25.6% and 20.5% in carbapenem recipients and non-recipients, respectively. A correlation between initial carbapenem use and increased in-hospital mortality was discerned in the unadjusted model (difference of 5.2%), but this association was not evident in the adjusted model (difference of 1.2%; 95%CI: -4.3,6.6; p=0.66).

Conclusions

Initial antibiotic therapy with carbapenems was noted in over half of the sepsis patients in Japanese ICUs. In-hospital mortality was not associated with the use of carbapenems.

## Introduction

Carbapenems have been widely used to treat bacterial infections in recent years. Studies from Latin American countries (2008 to 2009), Poland (2014 to 2015), and Japan (2011) indicate that the usage rate of carbapenems was approximately 20% [1-3]. A study from the United States indicated that carbapenems were prescribed 11.2% between 2004-2014 [4]. Furthermore, this usage trend has consistently grown over time. Longitudinal data shows an increase in Europe from 76 defined daily doses (DDD) in 2001 to 216 DDD in 2011. Similarly, in Germany, there was a rise from 76 DDD in 2001 to 250 DDD in 2015, while intensive care units (ICU) in Switzerland recorded an uptick from 114 DDD in 2009 to 156 DDD in 2018 [5-7].

However, carbapenems are typically considered last-resort antibiotics. The escalating use of these drugs has raised concerns about the rise in carbapenem-resistant bacteria [8-10]. Consequently, antimicrobial stewardship, which includes a carbapenem-sparing strategy, has been emphasized as crucial in combating antimicrobial resistance and reducing healthcare costs [11-13]. Further, implementing a carbapenem-sparing strategy in sepsis therapy could reduce costs without negatively impacting patient outcomes [14,15].

Finding a balance between the utilization and restriction of carbapenems in initial sepsis therapy is crucial. However, there is limited documentation on carbapenem use in the initial antibiotic treatment for sepsis. This study aimed to illustrate the prevalence of carbapenem use in the initial antibiotic treatment of septic patients, the prognosis of those who received carbapenems, and the variation in carbapenem use across various institutions in Japanese ICUs.

## Materials and methods

Design and data source

This study was a post hoc descriptive analysis of the sepsis sub-study from the Focused Outcome Research on Emergency Care for Acute Respiratory Distress Syndrome, Sepsis, and Trauma (FORECAST) study. FORECAST was a multi-center, prospectively collected cohort study conducted in 59 ICUs across Japan from January 2016 to March 2017 [16]. The FORECAST sepsis study included adult patients (age ≥16 years) with severe sepsis or septic shock, as defined by the sepsis-2 criteria, who were admitted to the ICUs of the participating hospitals [17]. Patients with limitations on sustained life care or those in a post-cardiopulmonary arrest resuscitation status at sepsis diagnosis were excluded. These details are described in a previous article (UMIN-CTR ID: UMIN000019742) [16]. According to the sepsis-2 criteria proposed in 2001, sepsis was defined in the FORECAST study as the probable or documented presence of infection along with the systemic response to that infection [17]. Additionally, severe sepsis was defined as sepsis accompanied by sepsis-induced organ dysfunction or tissue hypoperfusion. Septic shock was characterized as sepsis-induced hypotension persisting despite adequate fluid resuscitation [17,18].

Participants

Patients were selected from the FORECAST study if they had available data on the use of initial antibiotic therapy for sepsis. No exclusion criteria were applied.

Variables

The baseline characteristics of the study population included demographics, site of infection, and laboratory tests at the time of sepsis diagnosis in the emergency department or intensive care unit. The study's exposure variable was whether or not a carbapenem was used as the initial antibiotic treatment. Intermediate variables encompassed dialysis, polymyxin B hemoperfusion, sivelestat sodium hydrate, intravenous immunoglobulin, antithrombin, and recombinant human soluble thrombomodulin. We also evaluated characteristics of the study hospitals, such as the type and volume of the hospital and the presence or absence of an infectious disease department. The proportion of carbapenem use in each ICU was documented for study hospitals that enrolled more than three patients in the FORECAST registry. Outcome variables for the study were in-hospital mortality, 28-day mortality, 28-day length of hospital stay, 28-day ventilator-free days, and 28-day ICU-free days.

Statistical analysis

Descriptive statistics comprised percentages for categorical variables and medians with interquartile ranges (IQR) for continuous variables, as not all variables followed normal distributions. The association between the initial use of carbapenems and in-hospital mortality was considered to vary across both the site of infection and the hospitals. The association was assessed using a generalized linear mixed-effects model, with adjustments for age and Sepsis-related Organ Failure Assessment (SOFA) score at presentation as fixed-effect confounders and the site of infection and the identification number of the treating hospital as random-effect confounders. The treatment effect was estimated as absolute risk differences with 95% confidence intervals. Statistical analyses were conducted using R 4.4.2 (R Foundation for Statistical Computing, Vienna, Austria), a language and environment for statistical computing.

## Results

Of the 1184 patients registered in the FORECAST sepsis study, 1140 were included, and 44 patients were excluded due to missing antibiotic use data. Of the included patients, 627 (55.0%) initially received carbapenem treatment.

The age, sex, Acute Physiology and Chronic Health Evaluation II (APACHE II), and SOFA scores were comparable between groups (Table 1). Patients who received carbapenems had a higher incidence of septic shock than those who didn't. The most common sites of infection were the abdomen for the carbapenem group and the respiratory system for the non-carbapenem group. The carbapenem group showed a higher prevalence of anti-methicillin-resistant Staphylococcus aureus antibiotic usage, whereas the non-carbapenem group was more frequently treated with penicillin and cephalosporin antibiotics (Table 2). Moreover, carbapenem recipients exhibited higher rates of vasopressor agent use and dialysis (Table 3).

**Table 1:** Baseline characteristics of the study population Continuous and nominal variables were described as median values with 25-75th interquartile range and count with percentage, respectively. ICU - intensive care unit; APACHE II - Acute Physiology and Chronic Health Evaluation II; SOFA - Sepsis-related Organ Failure Assessment

Baseline characteristics	Overall, n=1140	Carbapenem group, n=627 (55.0%)	Non-carbapenem group, n=513 (45.0%)
Age	73 (63,81)	72 (64,81)	73 (63,81)
Sex (male)	696 (61.1%)	380 (60.6%)	316 (61.6%)
Charlson Index	1 (0,2)	1 (0,2)	1 (0,2)
Infection site			
Abdominal	297 (26.1%)	182 (29.0%)	115 (22.4%)
Respiratory	353 (31.0%)	159 (25.4%)	194 (37.8%)
Urinary tract	213 (18.7%)	127 (20.3%)	86 (16.8%)
Skin/soft tissue	112 (9.8%)	75 (12.0%)	37 (7.2%)
Catheter	22 (1.9%)	11 (1.8%)	11 (2.1%)
Central nervous system	22 (1.9%)	10 (1.6%)	12 (2.3%)
Osteoarticular	21 (1.8%)	8 (1.3%)	13 (2.5%)
Endocardium	16 (1.4%)	8 (1.3%)	8 (1.6%)
Implant device	8 (0.7%)	3 (1.0%)	5 (0.1%)
Wound	12 (1.1%)	4 (0.6%)	8 (1.6%)
Other	64 (5.6%)	40 (6.4%)	24 (4.7%)
SOFA	9 (6,11)	9 (7,12)	8 (5,11)
APACHE II	22 (17,29)	23 (17,30)	22 (16,29)
Septic shock	717 (62.9%)	456 (72.7%)	261 (50.9%)
Time to antibiotics use (min)	101 (55,191)	99 (51,190)	107 (59,191)
De-escalation	644 (56.5%)	410(65.4%)	234(45.6%)
Facilities form			
University hospital	690 (60.5%)	376 (60.0%)	314 (61.2%)
Teaching hospital	432 (37.9%)	246 (39.2%)	186 (36.3%)
Other	18 (1.6%)	5 (0.8%)	13 (2.5%)
Number of hospital beds			
≤500	177 (15.5%)	99 (15.8%)	78 (15.2%)
501-1000	723 (63.4%)	385 (61.4%)	338 (65.9%)
≥1001	240 (21.1%)	143 (22.8%)	97 (18.9%)
Number of ICU beds per year			
≤10	355 (31.1%)	183 (29.2%)	172 (33.5%)
11-20	558 (48.9%)	313 (49.9%)	245 (47.8%)
≥21	221 (19.4%)	128 (20.4%)	93 (18.1%)
Annual ICU admissions (data of 2013)			
none	5 (0.4%)	3 (0.5%)	2 (0.4%)
≤500	210 (18.4%)	100 (15.9%)	110 (21.4%)
501-1000	610 (53.5%)	351 (56.0%)	259 (44.6%)
≥1001	309 (27.1%)	170 (27.1%)	139 (27.1%)
Tertiary emergency facility	981 (86.1%)	538 (85.8%)	443 (86.4%)
Department of infectious diseases	490 (43.0%)	282 (45.0%)	208 (40.5%)

**Table 2:** Antibiotics use other than carbapenem Reported counts (proportions) for categorical variables. Penicillin antibiotics were penicillin G, ampicillin, ampicillin/sulbactam, piperacillin, piperacillin/tazobactam. Cephalosporin antibiotics were cefazolin, cefotiam, ceftriaxone, sulbactam/cefoperazone, cefepime, cefcapene pivoxil and cefozopran. Aminoglycoside antibiotics were gentamicin and kanamycin. Quinolone antibiotics were ciprofloxacin, pazufloxacin, moxifloxacin, sitafloxacin, tosufloxacin, levofloxacin. Macrolide antibiotics were azithromycin and clarithromycin. The tetracycline antibiotic was minocycline. Anti-methicillin-resistant staphylococcus aureus (MRSA) drugs were vancomycin, daptomycin, linezolid and teicoplanin. Antifungal drugs were fluconazole, caspofungin, voriconazole, micafungin and liposomal amphotericin B. Antiviral drugs were acyclovir and foscarnet. Other antibiotics were metronidazole and sulfamethoxazole trimethoprim and clindamycin.

Antibiotics	Carbapenem group, n=627 (55.0%)	Non-carbapenem group, n=513 (45.0%)
Penicillin	31 (4.9%)	315 (61.4%)
Cephalosporin	15 (2.4%)	148 (28.9%)
Monobactam	0 (0%)	0 (0%)
Aminoglycoside	3 (0.5%)	5 (1.0%)
Quinolone	12 (1.9%)	7 (1.4%)
Macrolide	0 (0%)	0 (0%)
Tetracycline	2 (0.3%)	5 (1.0%)
Anti-MRSA drug	189 (30.1%)	81 (15.8%)
Antifungal drug	34 (5.4%)	17 (3.3%)
Antiviral drug	0 (0%)	3 (0.6%)
Other	40 (6.4%)	20 (3.9%)

**Table 3:** Treatment other than antibiotics IRRT - intermittent renal replacement therapy; CRRT - continuous renal replacement therapy; PMX - polymyxin B hemoperfusion; Sivelestat - sivelestat sodium hydrate; IVIg - intravenous immune globulin; AT - antithrombin; rhTM - recombinant human soluble thrombomodulin

Other treatment	Carbapenem group, n=627 (55.0%)	Non-carbapenem group, n=513 (45.0%)
Norepinephrine	473 (75.4%)	258 (50.3%)
IRRT	22 (3.5%)	13 (2.5%)
CRRT	199 (31.7%)	95 (18.5%)
PMX	76 (12.1%)	21 (4.1%)
Protease inhibitor	54 (8.6%)	28 (5.5%)
Sivelestat	21 (3.3%)	17 (3.3%)
IVIg	165 (26.3%)	50 (9.7%)
Corticosteroid	223 (35.6%)	110 (21.4%)
AT	149 (23.8%)	73 (14.2%)
rhTM	168 (26.8%)	67 (13.1%)

**Figure 1:**
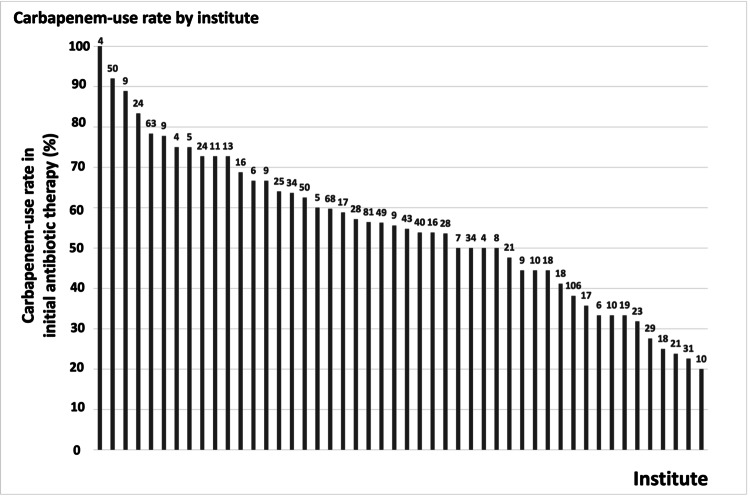
The proportion of carbapenem-use in each ICUs The numbers above each bar represent the number of registered cases. The study hospitals enrolled more than three patients in the FORECAST registry. ICU - intensive care unit; FORECAST - Focused Outcome Research on Emergency Care for Acute Respiratory Distress Syndrome, Sepsis, and Trauma

Out of the 59 participating ICUs, 48 enrolled more than three patients in the FORECAST registry. Among these ICUs, the prevalence of initial carbapenem use varied widely, showing a median of 55.2% (IQR: 43.6%-67.2%), ranging from 20.0% to 100.0% (Figure 1).

In-hospital mortality was noted in 158 (25.6%) of the patients who received carbapenems, compared to 101 (20.5%) of those who did not (Table 4). In the unadjusted model, initial carbapenem use was significantly associated with increased in-hospital mortality (difference of 5.2%; 95% CI: 0.2%-10.2%, p=0.04). However, this association was insignificant in the adjusted model (difference of 1.2%; 95% CI: -4.3%-6.6%, p=0.66). Initial carbapenem use wasn't significantly linked to 28-day mortality in either model. Yet, it correlated with longer 28-day hospital stays, fewer 28-day ventilator-free days, and 28-day ICU-free days in the unadjusted model but not in the adjusted model.

**Table 4:** Carbapenem use and patient prognosis In-hospital mortality, day 28 mortality were described using a percentage. Length of hospital stay, 28-day ventilator-free days and 28-day ICU-free days were described as mean (SD). The log-Poisson regression generalized linear mixed-effect model was used to assess the association between initial carbapenem use and hospital mortality after adjustment for the fixed effect variables (age and baseline SOFA score), and the random effects variables (institute and site of infection). ICU - intensive care unit; SOFA - Sepsis-related Organ Failure Assessment

Variables	Carbapenem group, n=627 (55.0%)	Non-carbapenem group, n=513 (45.0%)	Unadjusted difference (95%CI)	Adjusted difference (95%CI)
In-hospital mortality, n (%)	158/616 (25.6%)	101/493 (20.5%)	5.2 (0.2,10.2)	1.2 (-4.3,6.6)
28-day mortality, n (%)	127/612 (20.8%)	79/487 (16.2%)	4.5 (-0.1,9.2)	1.0 (-4.0,5.9)
Length of hospital stay, days	38 (42)	33 (35)	6 (1,10)	4 (-1,10)
28-day ventilator-free days	15 (11)	18 (12)	-2 (-3,-1)	-1 (-3,0)
28-day ICU-free days	15 (9)	18 (9)	-2 (-3,-1)	-1 (-2,0)

## Discussion

Our study revealed that over half of the patients with severe sepsis or septic shock in Japanese ICUs received carbapenems as their initial antibiotic therapy. Notably, there was considerable variation in this practice across institutions. After accounting for known confounding factors and clusters, carbapenem use did not correlate with improved in-hospital mortality rates.

In our examination of carbapenem usage among patients with severe sepsis or septic shock in Japanese ICUs, we found that 55% of patients were initially prescribed carbapenems. Previous guidelines, such as the 2016 Surviving Sepsis Campaign guidelines (SSCG) and the 2013 Japanese guidelines for managing sepsis, recommended the prompt initiation of empiric broad-spectrum therapy within one hour of diagnosis [19,20]. This recommendation might have contributed to the high rate of initial carbapenem use. Factors like limited awareness of antimicrobial resistance and inadequate promotion of antimicrobial stewardship practices in Japan may have influenced the preference for carbapenems in initial sepsis treatment [21-23]. 

Furthermore, the results of our investigation revealed no statistically significant disparity in mortality rates between the administration of carbapenem and alternative antibiotics. This aligns with the findings of other studies, which did not show significant differences in clinical efficacy, safety outcomes, and mortality rates when comparing carbapenems with alternative antibiotics [15,24,25]. Thus, evidence supporting carbapenems' superiority in clinical outcomes in sepsis treatment is scant. Interestingly, our study underscores the inter-institutional disparity in using carbapenems as an initial therapy. While the reasons for such differences remain unclear, prior studies have suggested that antibiotic use patterns can vary based on geographical location, country, and healthcare setting [26-28]. A multicenter survey conducted in North America indicated that several factors, such as facility size, number of ICU beds, use of non-carbapenem beta-lactams, and specific stewardship strategies, might influence the rate of carbapenem use [29]. In a survey of antibiotic prescribing practices in Japan, university hospitals and open ICUs exhibited significantly higher carbapenem use [3]. The elevated rate of carbapenem prescriptions in certain institutions might be influenced by factors like awareness of antimicrobial resistance, monitoring of antibiograms and antibacterial stewardship. However, these were not evaluated in our study. 

Our study has several limitations. The dataset did not specify the types of carbapenems administered. There was no evaluation of causative bacteria susceptibility or the prevalence of antimicrobial-resistant strains, limiting the assessment of antibiotic appropriateness. The study also did not analyze the dosage and duration of antimicrobial use, carbapenem administration technique, and length of stay in the hospital and ICU at the time of antibiotic therapy. Being retrospective, it did not evaluate causal relationships, and patient characteristics were not examined in detail. Follow-ups post-hospital discharge were not conducted. Although there is wide variability in carbapenem usage among institutions, there was no evaluation of antimicrobial stewardship programs or level of care. Moreover, the selection of facilities from the FORECAST study may limit the generalizability of our findings to the broader Japanese population. As a single-nation study, the external validity of our results for other countries is constrained.

## Conclusions

In Japanese ICUs, carbapenems were used as the initial antibiotic treatment for over half of the sepsis patients. However, the frequency of this practice varied across institutions. The use of carbapenems was not associated with improved in-hospital mortality rates after adjusting for known confounding factors.
